# 

**Published:** 1878-05

**Authors:** 


*CONTENTS.*

| | Page |
|---|---|
| Hacking Cough | 461 |
| Throat Ail | 462 |
| Croup | 462 |
| Bronchitis | 463 |
| Consumption | 464 |
| Spitting Blood | 465 |
| Imperfect Breathing | 466 |
| Necessity of Pure Air | 467 |
| How Blood is Purified | 469 |
| How Tubercles are Formed | 470 |
| How Tubercles Destroy | 471 |
| Important Fact | 473 |
| Important Advice | 475 |
| The Pulse Test | 477 |
| Acceleration of Breathing | 478 |
| The Spirometer | 479 |
| Latent Insanity | 480 |
| Healthfulness of Fruit | 482 |
| Malarial Diseases | 483 |
| Invalids | 483 |
| Health | 484 |
| The Mother's Remorse | 485 |

